# To thine own self be true: interoceptive accuracy and interpersonal problems

**DOI:** 10.1186/s40479-021-00175-5

**Published:** 2022-02-01

**Authors:** Wendy D’Andrea, Nadia Nieves, Treva Van Cleave

**Affiliations:** 1 The New School for Social Research, 80 Fifth Avenue, New York City, NY 10011 USA; 2 Mount Sinai World Trade Center Mental Health Program, 1468 Madison Ave, New York, NY 10029 USA

**Keywords:** Interoception, Interpersonal problems, Borderline personality disorder, Vagal tone

## Abstract

**Background:**

Borderline Personality Disorder involves strong interpersonal disruptions, often associated with early maltreatment. However, the individual capacities which alter BPD-related interpersonal problems are unclear. Here, we examine two contributors to interpersonal functioning: interoceptive accuracy and parasympathetic activity. Interoceptive accuracy is the ability to correctly perceive body states, such as how quickly one’s heart is beating, and has been associated with emotional experience and various crucial social capacities. Similarly, parasympathetic activity is related to social processing and inhibition of impulses. As such, both may contribute to BPD interpersonal symptoms, albeit different types of interpersonal problems.

**Method:**

Sixty-five individuals completed the Inventory of Interpersonal Problems and the Millon Clinical Multiaxial Inventory followed by a task to assess interoceptive accuracy, the heart rate monitoring task, in which participants counted their heartbeats while concurrent physiological data was recorded; and an assessment of vagal tone, used as an index of regulatory flexibility.

**Results:**

Participants who reported poor interpersonal boundaries, consistent with borderline personality disorder styles, had worse interoception, whereas those high in aggression had lower vagal tone. Borderline personality symptoms overall were related to IA and significantly to vagal tone.

**Conclusions:**

These findings suggest that interoceptive accuracy is associated with interpersonal problems, where people are overly influenced or enmeshed with others, possibly to compensate for the absence of their physical and emotional awareness.

**Supplementary Information:**

The online version contains supplementary material available at 10.1186/s40479-021-00175-5.

## Background

Borderline Personality Disorder (BPD) has been characterized as having three core features: emotion dysregulation, unstable relationships, and altered or absent sense of self  [32]. Emotion dysregulation may lead to interpersonal conflicts [48] when individuals fail to inhibit aggression and hostility. However, this formulation leaves out the third key feature of BPD, lacking a clear self or identity [21], which may contribute to the interpersonal problem of interpersonal boundary diffusion, because in the absence of stable internal cues, individuals may rely excessively on others. Here, we 1) examine whether two different types of interpersonal problems, namely, over-reliance on others on the one hand, and aggression/hostility on the other, both contribute independently to BPD symptoms; and 2) to examine whether these interpersonal problems correspond to indices of poor inhibitory capacity and poor self-awareness.

Theories about self-knowledge have been tied to interpersonal capacities through the interoception literature. Theories of body awareness and the self suggest that the “self” is constructed in part by integrating information inside and outside of the body to create a representation of the individual’s internal world [43]. Inability to sense internal body states may contribute to issues in identity formation, self-awareness, and emotional awareness [35]. Perceiving the physiological state of the body, referred to as interoceptive accuracy (IA) is linked to intrapersonal constructs such as emotional awareness [2, 47], body ownership [8], and the construction of a narrative “I” [15, 23]. Poor intrapersonal awareness may be related to poor interpersonal boundaries [33] and difficulties evaluating and trusting one’s own emotions [19].

The hostility, aggression, and impulse control in BPD may be characterized as deficits in self-regulation, particularly inhibitory control. Inhibitory control is facilitated by the vagus nerve [46], indexed by Respiratory Sinus Arrhythmia (RSA) [9]. RSA reflects the extent of the parasympathetic control that the vagus nerve has over heart rate, and further exerts influence upon the prefrontal cortex, insula, and superior temporal gyrus, which relate to inhibition, body awareness, and social awareness, respectively [6]. Low RSA has been identified as a contributor to affect disinhibition [46] and social behavior [45].

### The present study

Both IA and RSA may be distinct but interdependent contributors to the interpersonal problems associated with BPD, because low self-awareness and self-regulatory capacity may both impact social processes in BPD, albeit by different routes. The purpose of the present study is to further delineate the relationship between IA and personality traits that contribute to the experience of interpersonal problems. Because people with BPD have been characterized as both numb [36] and with high sensitivity to emotional states [34], an examination of both dimensions of IA—that is, hyper- and hypo-awareness of bodily cues—may help to elucidate aspects of body awareness that contribute to emotion dysregulation as well as interpersonal problems.

As it is currently configured, the formula for assessing IA computes errors in heart rate perception *independent of the direction of those errors* [40]. In contemporary IA measurement, people who fail to perceive heartbeats that are occurring are not distinguished from those who erroneously perceive their heart as pounding. This distinction may map on to clinically salient characteristics, as the former may relate more closely to numbing, and the latter may correspond to anxiety sensitivity. In contrast with studies that collapse over- and under-estimation, we examine over- and under-estimation separately. Specifically, we examine whether personality features characterized by inability to maintain boundaries would correspond to low ability to detect bodily signals, reasoning that individuals who cannot detect their own internal states may rely upon information from others. Similarly, we hypothesize that RSA will be related to BPD symptoms, particularly aggression and hostility.

## Method

### Participants

Participants ages 18 and older were recruited through community and university advertisements and compensated by research credits or $20. (See Table 1 for descriptives). Participants with known cardiac anomalies affecting the rhythm of the heart (pacemaker, arrhythmias) were screened out.

**Table 1:** Demographics and clinical characteristics

	M (SD)	N (%)
Race		
White or Caucasian		30 (45%)
Black		19 (28%)
Hispanic/Latino		17 (25%)
Asian		8 (12%)
IIP		
Domineering	5.9 (2.3)	11 (17.2%)
Vindictive	6.4 (2.6)	10 (15.6%)
Cold	7.4 (3.9)	17 (26.6%)
Socially Avoidant	8 (4.8)	18 (28%)
Assertive	7.9 (4.6)	16 (25%)
Exploitable	7.5 (3.7)	13 (20.3%)
Overly Nurturing	8.3 (3.8)	24 (37.5%)
Intrusive	6.5 (3.3)	19 (29.7%)
MCMI		
Borderline	39 (31.2)	14 (21%)

Note: More than one race could be selected. *IIP* Inventory of Interpersonal Problems; *MCMI* Millon Clinical Multiaxial Inventory

### Materials

#### Demographics

In addition to age, race, education level, and gender, participants reported their estimation of physical health (1 = excellent, 5 = very poor) and number of physical health visits. Advertisements recruited on the basis of salient clinical and life-history features for part of a larger study from which these measures was drawn.

#### Interpersonal problems measure-short form

The *Inventory of Interpersonal Problems (IIP* [4, 28]*;)* is an inventory designed to identify domains of interpersonal distress. The 32-item short form was utilized [44]. Subscales are classified according to whether the individual is described as agentic (A+) vs. submissive (A-), or communal (C+) vs. separate (C-). Subscales on this assessment include domineering (A+), vindictive (A+/C-), cold (C-), intrusive (A+/C+), overly-nurturant (C+), socially avoidant (A−/C-), nonassertive (A-), and exploitable (A−/C+). IIP alpha levels range from .82 to .94 [28]. Here, we utilize two composites: one of the A- scales: socially avoidant, nonassertive, and exploitable (“IIP-poor boundaries”); and another of the A+ scales (“IIP-aggressive”). Both the A- and A+ subscales relate to BPD (e.g., Pilkonis et al., 1996).

#### Personality disorder symptoms measure

The *Millon Clinical Multiaxial Inventory (MCMI*) is a self-report, 175-item inventory rated on a true or false scale [38]. The MCMI subscales are designed to assess clinically-significant personality pathology. Clinical significance is indicated in base rate adjusted scores greater than 75. Here, we focus on the Borderline subscale (*alpha* = .85).

Other measures. Other questionnaires (reported upon separately) on trauma history and symptoms were also collected. In this investigation, the anxiety subscale of the Brief Symptom Inventory (Derogatis, [17]) was included as potential confound.

#### Interoceptive accuracy and cardiac measures

Interoceptive Accuracy was determined by measuring heart activity with EKG sensors placed on the forearms and ankle using ANSAR electrodes and hardware and sampled at 250 Hz (Ansar Group, Philadelphia, PA) asking participants to report their perceived heart rate in two trials consistent with the instructions provided by Schandry [41]. Test-retest reliability of resting HRV measures has interclass correlations ranging from .74–.98 (Guijt, Sluiter, & Frings-Dresen, [24], demonstrating good test re-test reliability [11, 26]. IA has recently been shown to measure a relatively stable characteristic [20]. Collection and analysis of cardiac measures followed guidelines by the Society for Psychophysiological Research [9].

### Procedure

After consenting, participants completed questionnaires, followed by a resting baseline where heart rate was measured for 2 min, the epoch to accurately assess resting RSA (Berntson et al., 1993). They were then asked to count the number of heartbeats they experienced while actual heart rate was recorded in two trials, lasting 30s and 60s. All measures were taken when participants were in a seated position, at variable timepoints between 10 am and 4 pm.

#### Data reduction and analysis

The IA score is computed using the absolute value of the difference between actual and estimated beats (|Actual HR – Estimated HR|/Actual HR) [41], where lower scores represent more accuracy. This calculation controls for heart rate (i.e., perhaps a faster heartbeat is easier to detect); the absolute value provides information interoceptive accuracy regardless of over- vs. under-estimation of heart rate. As our goal was to examine errors in over- and under-estimation, we examined the IA score without the absolute value, as well. Numbers with the absolute value are presented for comparison’s sake.

The raw ECG signal and IBIs were manually inspected and corrected with midbeat correction, and RSA was calculated using MindWare Tech’s using Fourier transformation in the high-frequency range (0.15–0.4 Hz). Detrending was not utilized for heartbeat accuracy measures but was employed for RSA computation.

Personality variables were used as the dependent variables, whereas IA and RSA were used as independent variables. Analyses were bootstrapped 5000 times.

## Results

### Descriptives

Sixty-seven participants (49 females, 16 males, 0 trans-identified persons) ages 18–62 (M = 28.5, SD = 10.6) completed the study. Two participants had incomplete IA data due to computer errors and were not utilized in any analyses. All remaining participants has complete data and physiological values within the expected range, with no multivariate outliers. Mean ratings of physical health were between good and excellent (M = 1.88, SD = .74), and the average number of doctor’s visits in the past year was 4 (SD = 4.5). Five participants reported health problems that could impact cardiovascular health (*n* = 5, hypertension; *n* = 1 asthma). Self-reported physical health was not correlated with resting HR (*r* = −.07, *p* = .59), resting RSA (*r* = −.01, *p* = .59), or interoception (*r* = −.09, *p* = .47). Number of doctor’s visits was not associated with resting HR (*r* = .17, *p* = .21), resting RSA (*r* = −.14, *p* = .32), or interoception (*r* = −.19, *p* = .16). Resting RSA was higher in females (M = 6.54, SD = 1.28) than males (M = 5.20, SD = 1.94; *t* (54) = 2.41, *p* = .03) and in younger individuals (*r* = −.41, *p* = .001). Resting RSA was unrelated to race; resting HR was unrelated to age, race, or sex. There were no differences in IA between races or sexes, or any association with age. Because anxiety (measured with the BSI) or education level may play a role in heartbeat perception, we examined whether these findings were potentially due to these factors; neither was related to IA.

Average interoception scores were 0.35 (SD = .23; range: 0.03–.86); without the absolute value, M = 14.01 (SD = .32, range = −.71–.81); “raw” difference scores (e.g., uncorrected for heart rate and without absolute value) scores were 6.21 (SD = 20.01; range: − 87.02 – 126.36). Resting heart rate was 76.12 (SD = 12.92), and resting RSA was 6.06 (SD = 1.62). HR and RSA during the interoception task were 73.23 (SD = 13.20) and 6.65 (SD = 2.07), respectively. There was no significant change in HR or RSA from baseline to task (*t* (59) = 1.01, *p* = .32; *t* (59) = 1.73, *p* = .09). T-tests revealed no differences in trial-to-trial IA or physiology; thus, these trials were averaged to increase the stability of the measure.

Mean IIP and MCMI subscale values can be found in Table 1; on the IIP, 43 (66%) of participants had clinically-significant personality difficulties on at least one subscale. (Presented at the individual subscale level for sample descriptive purposes only.) Clinically-significant elevations in the MCMI Borderline subscales were found in 21% of the sample (*N* = 14). Half (*N* = 34) of participants did not have any clinically-significant personality pathology.

Borderline personality symptoms were correlated with both IIP-poor boundaries (*r* = .40, *p* < .001, CI = .18 -. 61) and IIP-aggressive (*r* = .68, *p* < .001, CI = .51–.80). These two subscales combined predicted BPD symptoms together, *F* (61,2) = 28.505, *p* < .001, Adj R^2^ = .47, and separately (*b* = .20, *p* = .04 for boundaries and *b* = .59, *p* < .001 for aggression).

### Hypothesis 1

**Fig. 1:**
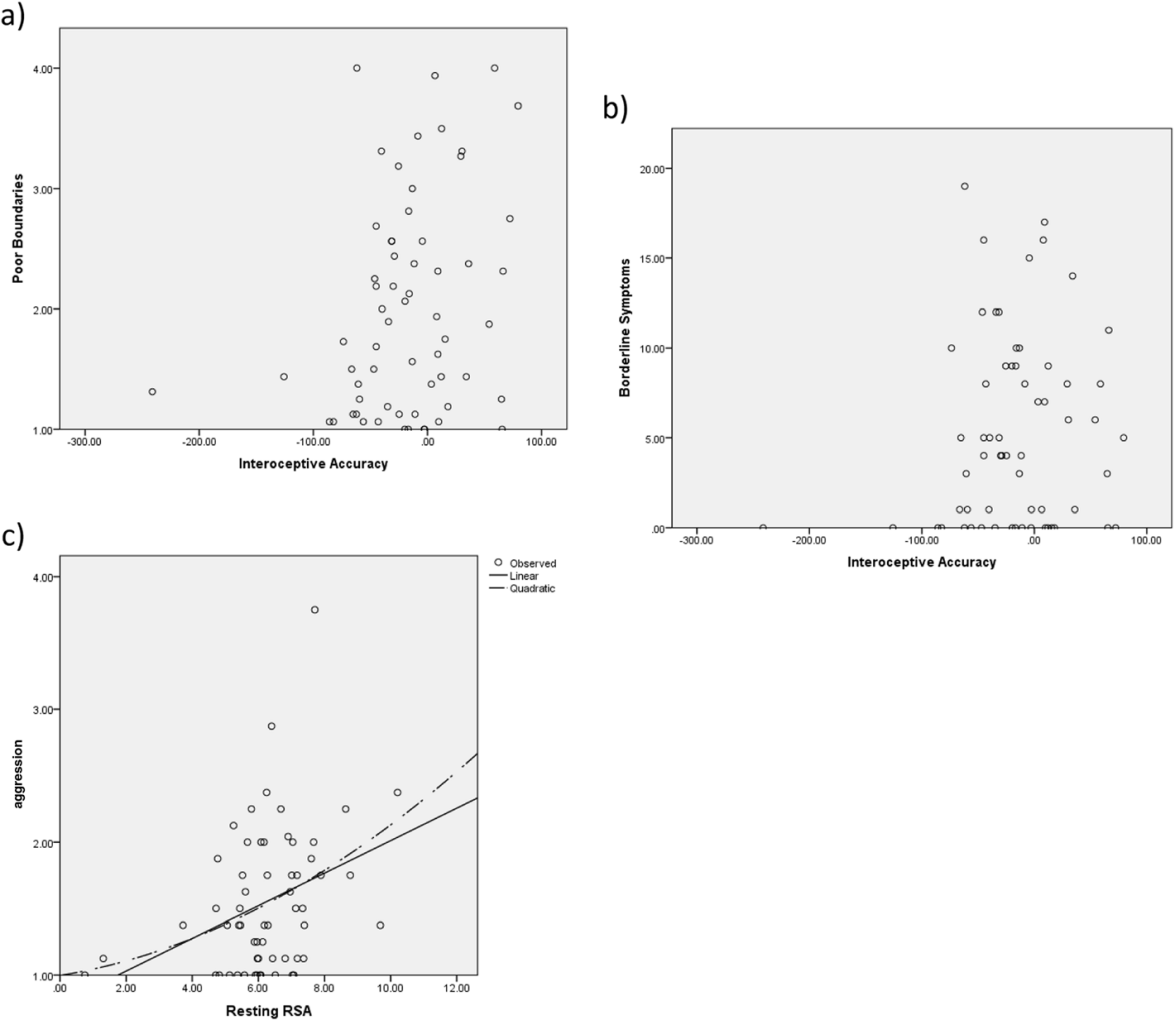
Relationships between Interoceptive Accuracy and Personality Traits. Scatterplots showing the relations between significant relationships: a) poor boundaries and interoceptive accuracy; b) borderline symptoms and interoceptive accuracy; and c) aggression and RSA

*Poor interoceptive accuracy will be related to interpersonal problems associated with poor interpersonal problems, and to BPD.* Under-estimation of heart rate was related to poor interpersonal boundaries (*r* = .30, *p* = .02), but not to aggressive behavior (*r* = .12, *p* = .42). BPD symptoms were related to interoceptive accuracy, but here, BPD symptoms were related to errors of either over- or under-estimation (*r* = .24, *p* = .04). Figure 1 shows scatterplots depicting the relations between significant relationships. See Supplementary Table 1 for correlations between poor boundaries and aggressive IIP subscales and interoception task scores.

### Hypothesis 2

*RSA will be related to interpersonal problems associated with aggression and BPD.* While RSA was related to interpersonal problems related to aggression (*r =* .35*, p =* .007*)* and BPD symptoms (*r =* .27, *p =* .04), the direction was opposite the predicted direction: higher RSA was related to more symptom reporting.

### Post-hoc analyses

Because the positive correlation between RSA and BPD symptoms was unexpected, we examined whether this relationship was linear. Indeed, Kogan et al. [30] found that RSA has a quadratic relationship to social traits, such that too high or too low RSA impairs social functioning. Thus, relations between personality and physiology were assessed using curve fitting for linear and quadratic analysis with confidence intervals bootstrapped 5000 times. Where quadratic analyses are significant, they are assumed to be a better fit of the data than linear analysis [37]. BPD symptoms were quadratically related to RSA, with low and high RSA related to higher BPD symptoms, *B* = 1.11, *p* = .013.

## Discussion

This study examined the contribution of interoceptive accuracy to interpersonal problems relevant to borderline personality symptoms: difficulties with maintaining boundaries with others and aggression. As hypothesized, we found that people who had trouble maintaining boundaries with others, that is, people who could not assert themselves or were overly accommodating, systematically under-perceived their heart rate. This finding was consistent with a trend towards a relationship of poor IA and higher BPD symptoms, suggesting that some of the diffuse boundaries occurring in BPD may be associated with an attenuated ability to notice one’s own states. Given that these personality problems are associated with low agency but high communality with others [27], we suggest that individuals who are unable to assert themselves or set boundaries in interpersonal situations may struggle because of difficulty accessing their own internal states. In the absence of clarity about one’s self, some individuals may become reliant on others. This interpretation is consistent with mentalizing [22] and mentalization-based psychotherapies for BPD [5].

Aggression was not associated with interoceptive accuracy. Instead, such interpersonal problems were associated with low vagal tone, consistent with the vagus’ role in impulse inhibition. Given that BPD symptoms are related to both aggression and boundarylessness, it follows that both vagal tone and interoception would contribute to BPD symptoms.

We note our limitations: first, only two trials of IA were recorded to avoid participant burden, as other measures were collected during this study. Fewer trials may lead to noise in the data, obscuring findings; noise is less likely to lead to false positives. RSA was measured for a short epoch which was sufficient for recording RSA and is comparable with gold-standard 5 min readings (see, for example, [3, 39]) but may be less reflective of overall trait vagal tone. We also note the sampling rate was not optimal for RSA, and, as these data were collected contemporaneously with the release of new guidelines in RSA reporting, not all recommended contributors to RSA were collected. Participants’ health problems may create difficulties with accurate physiological collection, but as health problems are common particularly in samples with psychological distress, these participants’ presence may reduce data reliability while improving generalizability. Some other important variables, such as smoking and alcohol use, should be collected in future work.

These data are enhanced by the presence of clinical-level personality symptoms despite general community and university recruitment, by convergent findings across measures, and by the presence of a racially-diverse sample. However, stronger findings may have emerged with an exclusively treatment-seeking group, and future studies should use clinical interviews. Some findings which approached significance should be considered for follow-up. Future studies examining the role of interoception in personality would also benefit from examining expectancy biases, and from parsing interoceptive accuracy from interoceptive awareness, conceptually and experimentally. Other constructs such as mindfulness and alexithymia, anxiety sensitivity, and experiential avoidance may contribute to this mixed self-report and observational approach.

## Conclusion

In sum, interpersonal problems feature prominently in psychotherapy [14], and may significantly impact well-being [25]. Individuals who are unaware of their own bodily signals may be unable to differentiate safe from predatory people, may exhibit fearful behavior in social situations, or may avoid social contact altogether. These findings suggest that therapeutic techniques which focus on becoming more aware of bodily sensations and signals, such as dynamic therapy [1, 7, 31], interoceptive exposure [16, 29, 42], and mindfulness [10, 12, 13, 18] may concurrently change interpersonal and interoceptive difficulties.

## Supplementary Information

**Additional file 1: Supplementary Table 1.** Relationship between personality and interoceptive accuracy.

## Data Availability

The datasets analyzed in the current study are available from the corresponding author on reasonable request.
