# Supplementary material for: To thine own self be true: interoceptive accuracy and interpersonal problems
Source: Borderline Personal Disord Emot Dysregul. 2022 Feb 1;9:6. doi: 10.1186/s40479-021-00175-5 (PMC8805262; doi:10.1186/s40479-021-00175-5)
Supplement: Supplementary file 1 — Additional file 1: Supplementary Table 1. Relationship between personality and interoceptive accuracy. [file 40479_2021_175_MOESM1_ESM.docx]

Supplementary Table 1. Relationship between personality and interoceptive accuracy.

|  |  |  |  |  |
| --- | --- | --- | --- | --- |
|  | Standard Formula | | Without Absolute Value | |
|  | *r* | *p* | *r* | *p* |
| IIP |  |  |  |  |
| Poor boundaries (A-) | .10 | .44 | .30 | .02 |
| Aggressive (A+) | -.04 | .75 | .12 | .42 |
| MCMI |  |  |  |  |
| Borderline | .24 | .04 | .20 | .11 |

Note: IIP = Inventory of Interpersonal Problems; MCMI = Millon

Clinical Multiaxial Inventory
